# AGEISM AT WORK: A THEORETICAL AND EMPIRICAL CONSIDERATION

**DOI:** 10.1093/geroni/igz038.2107

**Published:** 2019-11-08

**Authors:** Sarah A Vickerstaff, Mariska van der Horst, Debra A Street

**Affiliations:** 1 University of Kent, Canterbury, Kent, United Kingdom; 2 VU Amsterdam, Amsterdam, Netherlands; 3 State University of New York at Buffalo, Buffalo, New York, United States

## Abstract

Ageism at work is becoming an increasingly popular research topic. It has been claimed that ageism is a serious threat to the extending working lives agenda that is prevalent in many Western countries, including the US and many countries in the EU. In this symposium, we consider this concept from a variety of perspectives in order to better understand what ageism is as well as how it affects older workers. To get a better grip on the concept, Sarah Vickerstaff and Mariska van der Horst look at the intersectionality of this concept, by assessing its relationships with gender and disability. Clary Krekula uses the concept of age coding to look at how both age normality and ageism are constructed. Jaap Oude Mulders assesses how age-related stereotypes translate into employment preferences of employers. Hannah Swift et al., testing social psychological theories, uses results from two experiments to analyse the role of ageism in recruitment practices. This symposium is inter-disciplinary combining sociologists, social psychologists and gerontologists and further combines quantitative (Jaap Oude Mulders), qualitative (Clary Krekula, Sarah Vickerstaff, and Mariska van der Horst) and experimental (Hannah Swift) methods. Together, the papers in this symposium show various dimensions of the concept ‘ageism’ and how it affects older workers in three European countries: Sweden, Netherlands, and the United Kingdom.

